# Liberating an EFL teacher as an activist teacher professional identity in MA TESOL classrooms

**DOI:** 10.1186/s40862-022-00172-3

**Published:** 2023-01-16

**Authors:** Nesreen Alzhrani

**Affiliations:** English Language Institute (ELI), King Abdulaziz University, Jeddah, Saudi Arabia

**Keywords:** Continuous professional development, Practitioner enquiry, EFL teachers, An activist teacher professional identity, TESOL education

## Abstract

This study introduces a new model of practitioner research to develop an activist teacher professional identity for student-teachers and to enhance their understanding of the theory and practice of continuous professional development (CPD). The participants were ten female student-teachers enrolled in an MA program for Teaching English to Speakers of Other Languages (TESOL) at a university in Saudi Arabia. In this intervention, student-teachers constructed their version of CPD. Then they subsequently reflected on their definition of CPD and the factors contributing to its success. Qualitative research methods were used to collect data, including document analysis and individual structured interviews. The study sought to discover how the enquiry intervention improved student-teachers’ grasp of CPD theory and practice and contributed to the construction of their activist teacher professional identity. The findings may encourage the development and implementation of new forms of practitioner enquiry in MA programs to support the construction of different notions of professional identity found in the literature.

## Introduction

In English-as-a-Foreign-Language (EFL), CPD is a means of promoting the quality of teaching (Vani, 2020). High-quality CPD may improve EFL teachers’ proficiency, which is reflected in students’ learning (Ravandpour, 2019). Most interventions adopted in CPD in the EFL context were recently driven by teachers’ active engagement and reflective practices. For example, Bayram & Bıkmaz (2021) explored the use of lesson study as a professional development tool for EFL teachers. The aim was to move teachers from passive approaches to professional development to more active approaches where teachers can practice reflection. They found that this professional development module was an excellent approach to conducting quality reflection and systematically observing themselves and peers. Consistent with Bayram & Bıkmaz (2021) results, Yalcin Arslan (2019) found that EFL teachers’ professional growth and confidence are enhanced through observation and reflection. Uştuk & De Costa (2021) reported similar results. They concluded that interactive approaches to CPD such as a lesson study fostered reflective practice, enhanced collaborative levels, and boosted teachers’ agency. Afshar & Doosti (2022) found that professional development courses that are organized and arranged by administrators and consultants are not effective enough in meeting teachers’ needs. Instead, they argued that teachers’ needs and the methods through which the content of the professional development is delivered should be considered. Therefore, they claimed that to plan adequate professional development courses, teachers should select the course content and use interactive methods in content delivery, such as collaboration and collegiality among teachers. Zein (2017) observed that targeting EFL teachers’ needs in professional development may broaden their instructional and pedagogical knowledge and self-efficacy. He also asserted that active engagement in collaborative activities and reflective practices would improve teachers’ opportunities to link theory to practice. In all this, research suggests that active engagement based on reflective practice and teachers’ collaboration would improve the effectiveness of a CPD course. In the following section, I discuss CPD in the Saudi context.

In Saudi Arabia, CPD courses depend on top-down policies, meaning that local or international agents organize all CPD activities. According to Assalahi (2021), in response to the persistent request to improve the practice of teaching in schools, the education sector in Saudi Arabia has cared for the professional standards for teachers. However, professional development for EFL teachers in Saudi Arabia is regulated by socio-cultural factors and teaching and learning pedagogies found in the context (Al-Harbi & Ahmad, 2022). Therefore, teachers’ needs are not a priority but the institution’s (Ahmad & Shah, 2022). Professional development in the Saudi EFL context is conducted following a clear and organized agenda to meet institutions’ requirements (Ahmad & Shah, 2022). For example, locally, we have the National Centre for the Professional and Educational Development (NCTED), established in 2019 by the Ministry of education and the Education and Training Evaluation Commission (ETEC) (Assalahi, 2021). Internationally, government-led courses are organized by the British Council, NILE, Cambridge Assessment, Pearson, and a Cambridge English Teacher (CET) (Al-Harbi & Ahmad, 2022; Ahmad & Shah, 2022).

Teachers’ communities of practice where teachers can gather and organize their agenda and regulation for CPD courses are absent. Now, these communities have become a requirement in the Saudi EFL context. This requirement is supported by Alshumaimeri & Almohaisen (2017). They argued for teachers to be engaged in the planning and organization of CPD courses if we expect qualified and practical CPD courses. As such, this qualitative study used practitioner enquiry principles to encourage ten female student-teachers to design their version of CPD courses and evaluate other CPD courses during their MA TESOL in a course called Professional Development of English Language Teachers taught by the author in 2020. The author hoped that such a practice of CPD course design and evaluation might assist in broadening student-teachers’ understanding of CPD theory and practice and constructing an activist teacher professional identity defined by Sachs (2001). This notion of identity is developed to support teachers in creating learning communities in which they share knowledge and act as active members in developing innovative forms of CPD courses. These CPD courses are liberated from the bureaucratic dominance of agents and organizations.

This research study sought to explore student-teachers’ CPD courses design, evaluation projects, and structured interviews to answer the following research questions:

1- How do practitioner enquiry principles broaden student-teachers’ understanding of CPD theory and practice? This understanding includes:

1. Student-teachers’ definition of CPD.
2. Their perceptions of the factors that constitute an effective CPD course.

## Theoretical framework

### Practitioner enquiry

Research enquiry has been commonly encountered and supported in teacher education programs. According to Cochran-Smitha et al., (2012), combining enquiry elements in teacher education is acceptable to train teachers to be lifelong learners. It acts as a universal development informed by unique belief systems, where there is a shared commitment to enquiry by teachers and practitioners to communicate developed actions (Hulme et al., 2009). For this reason, practitioner research enquiry also serves as an essential tool to enhance teacher agency (Newman & Leggett, 2019).

Practitioner enquiry is defined by terms such as ‘action’, ‘collaborative’, ‘narrative’, ‘pedagogy’, ‘participatory’, ‘autobiographical’, ‘reflexive’, and ‘critical’, which are integrated within noun phrases that describe the organised investigation of educational ‘research’, ‘enquiry’, ‘scholarship’, and ‘study’ (Cochran-Smitha & Lytleb, 2004, p. 603–604). Organised investigation is accomplished by an individual referred to as a ‘teacher’, ‘practitioner’, ‘teacher educator’, ‘participant’, or ‘self’ (Cochran-Smitha & Lytleb, 2004, p. 603–604). The investigative method provides a synthesised and coherent encounter for student-teachers that permits active engagement inside the classroom, making it a vital and innovative experience (Hulse & Hulme, 2012). It is also defined as an approach in which teachers can enhance their practice by promoting specialist knowledge about their teaching (Furlong, 2011). In addition, Cochran-Smitha & Lytleb (2004) stated that it is a means of enquiry that aims at reinforcing knowledge in practice by focusing on practitioners’ knowledge gains.

Murray (1992) found that teachers’ in-service education focuses on teaching knowledge, skills, and strategies to alter techniques. However, in Practitioner Based Enquiry (PBE), teachers are asked to critically examine and reflect on their daily working conditions and routines (Murray, 1992). As such, enquiry embraces self-reflective investigation of the practitioner’s schemes via direct involvement (McIntyre, 2006). In PBE, the emphasis of enquiry shifts from being fixed and arranged in the institutional setting, such as with essay writing and assignments, to collections of issues that educators encounter (Murray, 1992). From a practitioner enquiry standpoint, teachers are expected to understand and shape knowledge and education that underlines their ways of knowing (Cochran-Smitha & Lytleb, 2004). They attend to classroom concerns, develop teaching practices, and promote an appealing and supportive school environment that cultivates and encourages student learning (Stevens et al., 2016). Therefore, teachers who conduct PBE are equipped with advanced knowledge about what they wish to change, replicate, and remove in their daily working routine to achieve definitive personal goals (Murray, 1992).

According to Lunt & Shaw (2015), practitioner research comprises the following aspects: data collection, data reflection, framing intentions, and outcomes, identifying applicable advantages for professionals or originators, administering enquiry and concentrating the research on individual and group practices. Adopting such an enquiry viewpoint may facilitate the development of teachers’ understanding of the highly controversial relationship between practice, theory, and policy in teaching (Dickson, 2011). It also entails acquiring research skills and venturing away from their current occupational role (Murray, 1992). The critical concept of such a process is the structured reflection to improve perceptual and cognitive ability while actively engaging in a systematic integration of inspection, self-dialogue, judgment, and understanding of the research methodologies’ obligations (Murray, 1992). This systematic enquiry practice emphasises two critical points: teacher autonomy and knowledge-sharing with the public (Hall, 2009). Accordingly, teachers express their knowledge and understanding of teaching practices, providing confidence, and fostering engagement with methods and approaches that reflect their future pedagogical and practice decisions (Hall, 2009). Consequently, teachers are empowered to create and contribute their mastery of skills within schools and construct a more extensive body of knowledge by generating context-specific understanding (Torrance & Forde, 2017). These principles and merits of practitioner enquiry have led to the emergence of reflective practitioner designers of CPD courses based on two practice phases detailed in the methods section.

## An activist teacher professional identity

The primary reason for developing reflective practitioner designers of CPD courses was to provide opportunities for an activist teacher professional identity to emerge and allow the inclusion of cooperative teachers in CPD courses planning and designing. This identity was developed by Sachs (2001), who states that “equity and social justice” (p. 157) are two values forming the basic fabric of such an identity. As a result, she suggests that developing such a professional identity can aid parties or individuals in eliminating control over them and allow for flexibility and innovation in practice. Therefore, the author seeks to develop this identity in the field of CPD because, as stated by Kirkwood and Christie (2006), CPD often measures the effectiveness of conveying learning outcomes for students in a specific context rather than improving the professional knowledge of teachers. This CPD perspective is shaped by a system of bureaucratic and managerial practices and a lack of socially democratic views, which have collectively limited teachers’ autonomy and agency in CPD (Kennedy, 2014). It rewards the teacher for compliance rather than the agency, resulting in CPD courses focused on market needs and accountability demands (Sachs, 2001; Kennedy, 2014), making it challenging to work in a fast, changing, uncertain and vulnerable environment while maintaining a clear sense of what it means to be an educator in modern society (Sachs, 2001). Therefore, to shift the power of organizations and agencies over CPD and create a critical shift in teachers’ commitment and performance to CPD theory and practice, reflective practitioner designers of CPD courses were developed.

In conducting reflective practitioner designers of CPD courses, teachers actively engage in CPD planning to understand CPD theories and practice. They experience CPD through drafting and initiation CPD courses according to their context’s policies, culture, values, and needs. Therefore, they experiment with CPD principles not through the lens of a trainee but through that of a program manager or course trainer. In doing so, the teacher educator strives to create a democratic perspective of professionalism that leads to an activist teacher professional identity, in which teachers are active learners striving to develop practice communities (Sachs, 2001). The present study aspired to prepare teachers proficient in initiating and implementing their version of CPD courses and building learning communities in their contexts.

## Methods

Inductive content analysis guided this study’s data collection and analysis. It is used to describe how participants construct the meaning of the phenomena under study using text-based data (Vears & Gillam, 2022). The phenomenon under study is student-teachers using reflective practitioner designers of CPD courses guided by practitioner enquiry principles to understand CPD theory and practice. The main reason for student-teachers involvement in CPD course design and evaluation is to construct Sachs’s (2001) notion of an activist teacher professional identity in EFL teaching. The text-based data are from their CPD-designed courses, their evaluation of CPD courses, reflections, and structured interviews. The research questions were open and focused on the effect of practitioner enquiry tools in enhancing student-teachers’ understanding of CPD theory and practice, as well as the effect of reflective practitioner designers of CPD courses on the construction of an activist teacher professional identity defined by Sachs (2001). The following section explains the text-based data in detail.

## Procedure

### Reflective practitioner designers of CPD courses

The author was looking for a new research enquiry approach beyond simple classroom problems and reflections upon language pedagogy and practice to engage teachers in broader policy planning activities. This engagement would enhance and broaden student-teachers’ knowledge of CPD courses theory and practice to meet their professional needs. Thus, guided by the principles of enquiry research outlined in the literature review section, student-teachers in this study experienced the role of CPD planning through the lenses of a program manager or course trainer to connect theory to practice via reflective practitioner designers CPD courses. This developed strand of enquiry is based on two phases. In the first phase, student-teachers plan a CPD course to address a teaching dilemma of their choice. To plan their CPD courses, they use concepts and models derived from the following CPD theories: first, aspects of professional learning which were personal, social, and occupational (Bell & Gilbert, 1996). They choose which aspect they want to address in their CPD course. These aspects are the teacher’s personality, which includes his/her beliefs, values, ​​and experiences, as well as the school community’s development as an essential aspect in providing opportunities for teamwork within one’s environment (Fraser et al., 2007). Finally, the occupational aspect linked theory to practice by providing evidence-based research to convince trainers of their effectiveness and usefulness in classrooms (Fraser et al., 2007).

Second, the pedagogy approach used to deliver the content of the CPD course. It is either a transformative practice following the constructive approach to CPD based on reflective learning pedagogy or a transmission practice following the behavioural approach to CPD based on the acquisition and structured learning pedagogy (Kennedy, 2014). The third is the sphere of action in which professional learning occurs (Fraser et al., 2007). This learning could be planned/formal in structured courses or incidental/informal in staffroom chats (Fraser et al., 2007). To ensure successful program implementation, they finalised their CPD courses with all the necessary tools (teaching material, worksheets, PowerPoint presentations, and evaluation methods).

**Fig. 1:**
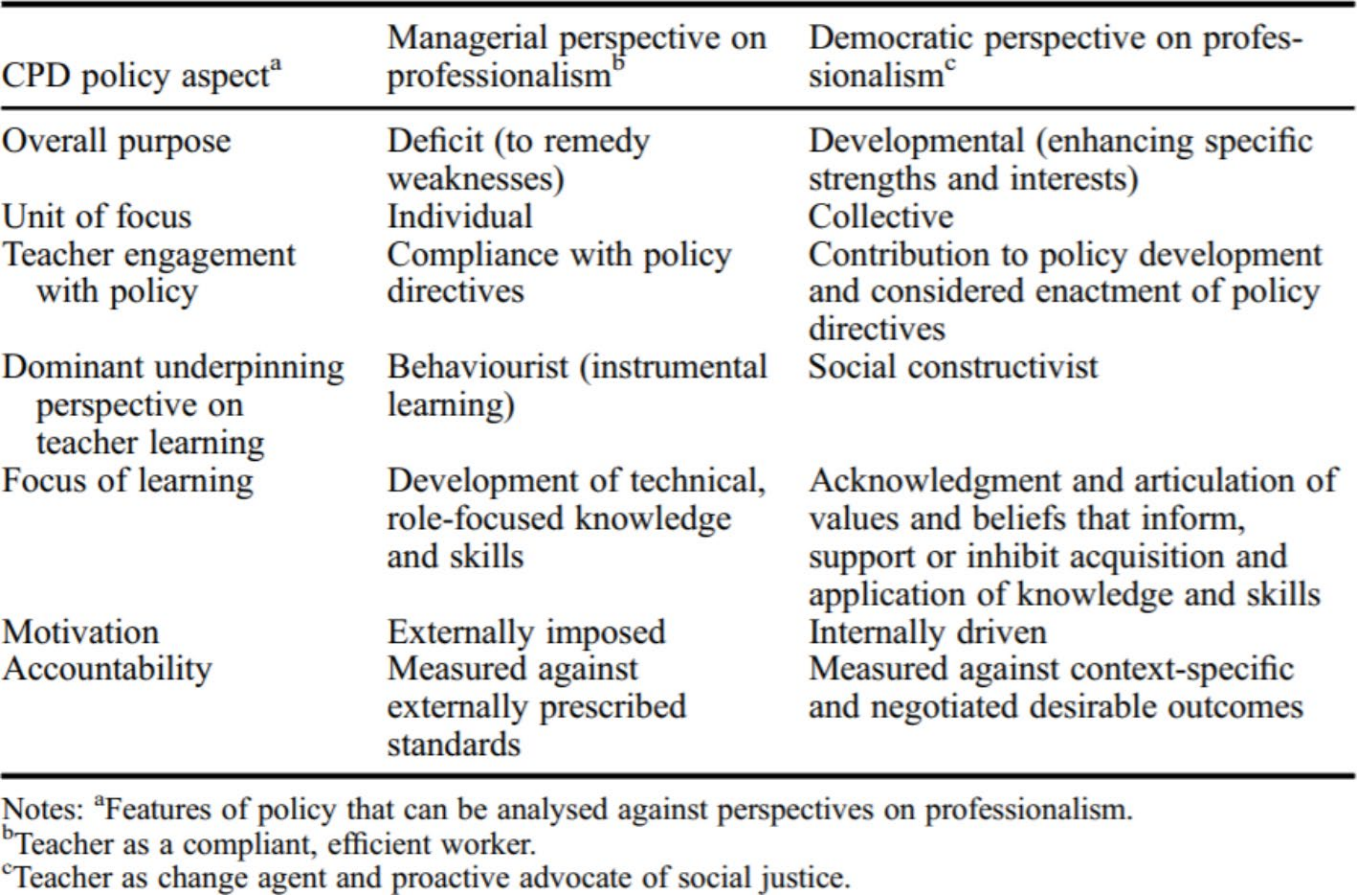
A guideline that teachers used to outline aspects of CPD policies against perspectives of professionalism. Adopted from Kennedy’s (2014, p. 965) analysis of CPD aspects that student-teachers used to guide their understanding of CPD in the second evaluation phase

In the second phase, student-teachers observed a CPD course using Kennedy’s (2014) (see Fig. 1) analysis of aspects of CPD policies against perspectives of professionalism. They analysed and deconstructed the elements that built the structure of the course through a critical evaluation of the course’s objectives, content, tools used, and teaching methods. Through this role, teachers outlined the policies, theories, and perspectives underpinning the attended CPD courses and how to improve them in the future. Finally, they reflected on the defining CPD from their perspective and explained the factors that constitute an effective CPD course.

Their understanding was measured by their ability to connect CPD theories to practice in choosing an appropriate CPD model based on an appropriate CPD theory to address each teaching dilemma and defining CPD and its success factors.

### Data collection

Data collection methods were based on document analysis and structured interviews, which started after completing the practitioner reflection intervention. First, the documents consisted of student-teachers’ CPD courses, evaluations of CPD courses, and reflections. CPD courses and evaluations for the CPD courses were completed in the last week of February 2020 and in May 2020, respectively. In the first phase, the design phase, student-teachers’ CPD courses, were examined to explore the participants’ choices of the teaching dilemma, model of CPD, and choice of CPD theory in designing their courses (see Table 1). The author examined the projects to uncover students-teachers’ understanding of CPD theory and practice and explore their ability to plan and organize a CPD course. The second phase, evaluations phase, and reflection of CPD courses were used to investigate the participants’ definition of CPD and the factors that constitute an effective CPD course.

These two phases constitute the intervention organized by the author: reflective practitioner designer of CPD courses. They answered the research questions by providing data on how reflective practitioner designers of CPD courses guided by practitioner enquiry principles enhanced student-teachers’ ability to act as course designers of CPD courses. In the first phase, they put CPD theory into practice by gathering information about the problem, choosing a solution, and designing and planning an appropriate course that meets their teaching needs and solves their teaching dilemmas.

In the second phase, they systematically observed their colleagues’ CPD courses and evaluated them. Therefore, they used their knowledge of CPD theory and practice from the first phase guided by Kennedy’s (2014) analysis of CPD aspects (see Fig. 1) to evaluate CPD courses in the second phase. After conducting the two phases, they are expected to understand CPD theory and practice thoroughly and reflect on the definition of CPD and factors affecting an effective CPD course.

Structured interviews were conducted to compare student-teachers’ answers in the evaluations and reflection documents of CPD courses versus their interviews regarding perspectives on CPD definition and effectiveness. The interviews were held three months after conducting their CPD courses designed by practitioner reflection. Each interview lasted approximately 20–25min and was conducted in English. The interviews centered on two open-ended questions asking for student-teachers’ definitions of CPD and factors that constitute an effective CPD course. All interviews were recorded and transcribed by the author and were around 6000 words in length for all ten interviews. The interviews were conducted via phone calls to adhere to social distancing regulations.

## Student-teachers

The student-teachers were ten Saudi female EFL student-teachers enrolled in an MA TESOL program in an English Language Institute (ELI) at a Saudi University. These student-teachers were chosen because they participated in CPD courses designed based on practitioner reflection in the last semester of 2020 and were the author’s students. Therefore, they were all well acquainted and had experience with CPD courses designed based on practitioner reflection. They voluntarily agreed to participate in this study. Their teaching experience ranged from five to thirteen years and their ages from twenty-eight to forty-one years old.

## Ethical considerations

The author taught the course intending to develop an activist teacher’s professional identity by immersing the student-teachers in CPD theory and practice. Student-teachers participation in the investigation began after they had finished the course, received their results, and had no additional classes with the author. Student-teachers were approached to participate in interviews and give access to their CPD projects and reflections, which constitute the two phases of the reflective practitioner designers of CPD courses, after the course. They were informed of the purpose of this study and signed written consent forms, which explained that they could withdraw from the study at any stage, that their interviews would be audio recorded, and that the information they shared would be anonymised. Ten out of eleven student-teachers agreed to participate in the interview and give access to their CPD projects. Student-teachers were also assured that the appropriate institutional authorities had approved the study. Approaching the student-teachers at the end of the course gave participants the freedom to decide whether to participate without causing any conflict of interest.

### Data analysis

The author used inductive content analysis to answer the research questions. This analysis process includes “data reduction, data grouping, and the formation of concepts that can be used to answer research questions” (Kyngäs, 2020, p. 14). The author used inductive content analysis to describe how student-teachers conceptualized the meaning of CPD and the factors contributing to successful CPD after conducting CPD courses designed by practitioner reflection. Another purpose of the data analysis was to describe how a CPD course designed by practitioner reflection could construct an activist teacher professional identity in an MA TESOL program.

The analysis process was as follows: first, the author coded the data collected from student-teachers’ CPD projects in the first phase into a table presenting their choices of CPD courses designed to grasp their understanding of the topic. Second, the author conducted the initial coding, which involved constant comparative analysis and aimed to identify similarities and differences for clarifying concepts and categories related to the topic under research (Chun Tie et al., 2019). This coding included data from the second phase: written reflections about the meaning of CPD, factors that constitute a successful CPD, and their evaluation of CPD courses into two categories based on the two research questions. Next, the recorded interviews were transcribed and compared with the ideas expressed in the written reflections and their evaluation of CPD courses. The author could observe a degree of consistency between the ideas expressed in the written reflections and those expressed through the interviews. Data were coded using student-teachers’ real names to keep track of responses and line by line, using the comment application available for Word document files. The tracking review comments were also used as referenced records to include the author’s comments on the data.

The initial codes were related to two major concept definitions of CPD, and factors associated with effective CPD courses. Thus, the focused coding was based on the author’s interpretation and understanding of the initial coded data (Chun Tie et al., 2019). It consisted of the following cluster of the initial codes: definition of CPD and factors of effective CPD. Under each cluster, there were codes related to the central cluster and other codes under the same cluster (see Table 2).

**Table 1:** EFL student-teachers’ professional development courses

Participant no.	Dilemma	Model of CPD	Capacity for professional autonomy in professional learning (Kennedy, 2005)	Sphere of action in (Fraser et al., 2007)
**1**	Teaching English in large classes	Group discussions and collaborative reflection	Transformative	Planned and incidental
**2**	EFL students’ speaking anxiety	Reflective writing	Transmission/ Transformative	Planned
**3**	Improve students’ reading skills	Action research	Transformative	Planned
**4**	Lack of technology integration	Coaching\monitoring model	Transmission	Planned
**5**	Providing feedback for students during classes	Action research	Transformative	Planned
**6**	Enhance students’ thinking skills	Action research	Transformative	Planned
**7**	Integrating technology in providing feedback to students	Training course (workshop)	Transmission	Planned
**8**	Assessing writing assignments for a classroom with many students	Training course (workshop) Reflective journals	Transmission Transformative	Planned
**9**	The use of multiple mice presentation in EFL collaborative learning classrooms	Training course (workshop)	Transmission	Planned
**10**	Bridge the gap between theory and practice in teaching grammar	Training course (workshop on the flipped classroom)	Transmission	Planned

**Table 2:** Coding process

Raw data	Initial code	Focused codes
1- In my opinion, a CPD program is a solution to an issue.	solution to an issue	
2- CPD should be planed based on a need in the institution. It should be chosen carefully to serve a purpose, improve professional skills and pedagogical knowledge of teachers and solve a problem and upraise the level of students.	needs of institution solve a problem improve students’ learning	
3- CPD programs train teachers to improve their performance which ultimately enhances the learning outcomes. It can target either teachers’ development or students’ achievement.	enhance teachers’ knowledge to improve students’ learning outcomes	Definition of CPD
	priority is to organizational objectives.	
4- A CPD program should be in line with the organizational objectives.	Related to institution’s needs- contextually oriented	
5- CPD programs should stem from the needs of a particular school or institution. It should have a strong relationship with the context where it is held.		

The data were sent to a colleague to verify the analysis, examine the meaning and interpretation, and review the steps followed in the data analysis. I was advised to remove coded data about institutions adapting to CPD demands under the working conditions of Covid-19 as it was beyond the scope of this research study. Finally, the author sent the data to the student-teachers to obtain their final approval of the reported findings.

## Results

Results are presented in three major sections: EFL student-teachers’ professional development courses, EFL student-teachers’ definition of CPD, and factors that constitute an effective CPD course. The first section displays a table showing the teaching dilemmas they identified and the type of CPD courses designed to resolve them. The second section elaborates on student-teachers’ understanding of the meaning of CPD after designing and evaluating a CPD course. The third section presents student-teachers’ perspectives on factors that constitute an effective CPD. Excerpts from interviews and reflections illustrate the second and third sections.

## EFL student-teachers’ professional development courses

Table 1 shows the teaching dilemmas to resolve as identified by the ten participants. These issues included: teaching English in large classes, EFL students’ speaking anxiety, improving students’ reading skills, lack of technological integration, providing feedback to students during classes, enhancing students’ thinking skills, integrating technology in providing feedback to students, assessing writing assignments for a classroom with a large number of students, the use of multiple mice presentation in EFL collaborative learning classrooms, and the gap between theory and practice in teaching grammar. They then chose a CPD model to inform their CPD courses. As the table illustrates, six participants decided to apply action research and reflective writing approaches to address their teaching dilemmas. Moreover, it is evident from the table that four participants selected workshops based on lecturing to tackle their identified teaching dilemmas. Concerning the capacity of autonomy allowed and sphere of action (Kennedy, 2005; Fraser et al., 2007), participants preferred transformative-based pedagogy in CPD over the transmission-based approach. Furthermore, the table demonstrates that knowledge acquisition in most CPD courses was based on planned action found in systematic procedures of enquiry of action research and reflective writing. Below is a discussion of participants’ responses to both reflection questions.

## EFL student-teachers’ definition of CPD

Teachers defined CPD as a tool that enhances teaching knowledge in content and pedagogy for EFL teaching. It should influence their pedagogy, improve students’ achievements, and meet the needs of organisations, institutions, stakeholders, and the community. They also defined CPD as a solution to teaching problems, a sequence of activities conducted within the course to improve teachers’ knowledge. Others observed CPD as a diagnostic tool that could improve practices and guide them to the best pedagogy in the EFL field. As student-teachers indicated (reflections):

A professional development program is a process that helps teachers to be successful and competent in their careers by improving their knowledge, skills, and their attitudes. Its ultimate goals are to increase students’ achievement by changing teachers’ practices inside the classroom.

Professional development program is a training program that can be in various forms, such as training workshops, communities of practices, monitoring, reflection, or action research.

The main aim of the CPD is to develop teachers’ or trainees’ pedagogical and content knowledge to maximize the benefit for teachers and to develop their career and skills to be more sufficient, with effective current teaching strategies.

A professional development program is any teacher training that improves teachers’ performance which ultimately enhances the learning outcomes.

(interview):

I believe that professional development is a well-designed plan that aims to give the chance to expand teachers’ skill.

It is a continuous practice. It is a process that improves teachers’ teaching and pedagogical knowledge.

However, this knowledge should be current and updated. In addition, CPD topics should be contextually oriented and derived from teachers’ needs in practice. Sociocultural, environmental, and contextual factors should be considered when planning CPD courses. They argued that not just any borrowed teaching method or model could work in the Saudi context. A few CPD practices do not work, such as cooperative teaching and placing students into groups. They shared that it was contradictory to ask them to teach students using strategies that were impossible to use in such crowded classrooms. (interview):

The program must achieve its aims and objectives and these objectives must be realistic and applicable in a way that suits our Saudi culture.

(reflections):

CPD should aim at expanding the knowledge and skills of a person or a group of people. However, the knowledge presented should consider teachers’ personal preference, and group preferences and match their objective.

In my opinion, a professional development program is effective when it is derived from the teachers’ context.

## Factors that constitute an effective CPD course

Student-teachers identified three main factors: the knowledge of the trainer or coach, CPD policy, and the practice and model of CPD. Student-teachers observed that the CPD trainers should be well trained to present their courses in a specific context. They observed that they should build their practice on adult learning theories and focus on conceptual knowledge as it contributes to lifelong skills. The knowledge presented should improve cognitive ability to understand how to teach. Further, the underlined theories of each pedagogical decision they make in the classroom, the knowledge presented should be transformative. Three student-teachers stated the following (interview):

A professional development program should be in-line with the adult learners’ characteristics. It is an important starting point, and it is better to target conceptual knowledge because it remains with the student-teachers for a longer period.

A professional development program could be effective due to the use of adult learning theories since all professionals are adults.

The program should be based on appropriate theories, such as adult learning theory and more teacher-driven approaches.

They also noted that CPD course presenters should have a deep knowledge of the area they are covering. They wanted the teacher trainers to know how to use hands-on activities and for trainees to implement what they learn within the course timeframe. The hands-on activities are essential in a CPD course. They should be designed to help teachers apply the learned knowledge during the CPD course. They stated that trainers should present their learning outcomes and objectives and pay attention to what they present, which should be relevant. Passion and empathy are two emotions that presenters need to show in courses. They should be honest and open about their experiences and share their thoughts with the trainees. They must also clarify the challenges and obstacles when planning and presenting courses, be mindful, and have substitutive plans, as indicated by student-teachers (reflections):

The trainer should be an expert in the area that the professional development program is given. They should know the audience and the skills they want to learn.

A trainer should be aware of the research-based evidence in the same context or a similar context. It is important to be cognizant of the fact that the better you know your context the more successful the professional development will be.

The content of a professional development program should be closely examined, and the trainer should possess excellent content knowledge, good presentation, and interpersonal skills to engage the audience.

Trainers should have concise outcomes and must have the proper activities to ensure the fulfilment of these outcomes. If there are challenges to carry out CPDs, they should be admitted working out better solutions and make them feasible as much as possible.

They also added that CPD is a high-demanding task that requires knowledge and skills to manage the course; thus, trainers should be well prepared for such a task to meet the desired outcomes. Their learning outcomes should be communicated to trainees, potentially engaging them to set their desired learning outcomes. Support should also be extended even after the CPD course as they argue for follow-up sessions with trainees to observe their understanding of the CPD content and evaluate their learning outcomes. The presenter must master skills, not only regarding the presentation’s content but also in its effective presentation and delivery. Trainers should have extensive knowledge of teaching, designing, and presenting workshops. Moreover, delivery methods should vary and not be lecture-dependent. Student-teachers also highlighted the importance of knowing how to run the workshop/training course and manage time wisely, considering the location and its facilities. The following example underscores this point (reflections):

Time and place play a vital role in the effectiveness of these training courses. Hence, it is important that the organizers of the professional development program consider the significant impact of time and place prior to organizing such activities in Saudi EFL institutions.

Any professional development program should have a focus, clear idea and very precise learning objectives. Models, timing, and venue should be carefully designed and chosen to serve and achieve the learning outcomes of the program.

Student-teachers were hoping for trainers who could increase their self-efficacy and passion for teaching. Concerning CPD policy, student-teachers mentioned some factors that should be considered when identifying CPD and its methods such as collaboration between teachers and trainers to identify their needs, goals, and teaching values. Student-teachers stated (reflections):

I think that the most effective professional development is the one that is built on a collaboration between a trainer and teachers from the very beginning. Teachers should be involved in the planning process since they are more aware of what is going on in the classroom.

Teachers’ perspectives should count to help them gain an obvious development in their pedagogical content knowledge, passion about their work, and notice a boost in their positive influence on their classroom practices.

Furthermore, they believed their freedom should be respected, and that course providers or managers should not force them to join CPD courses. It should be optional and based on teachers’ understanding of their needs. Student-teachers said, (interview):

Teachers must feel welcome to discuss, share their experiences and feel acknowledged for their time and effort.

[I] would encourage teachers to attend my school, but some of my colleagues refused to attend because the topic means nothing to them.

Student-teachers mentioned that, in some cases, they were deceived by the title of the CPD course and found that it had nothing to do with the topic they sought to learn. The following statements highlight this point (interview):

Sometimes, I read the title of the program, or the course and it is deceiving when you see that the title is very interesting and very attractive. But when you attend the program, you are not satisfied with what is being presented.

Our workplace cares only about the quantity, the number of professional development programs that are given, not their quality. So, you might find them offering redundant or repetitive ways of taking up teachers’ time and effort [that could have been spent] on something else. A teacher must prove that she has attended a specific number of programs.

In terms of the CPD model and practice, student-teachers observed that reflective writing is an effective learning method in a CPD course. They considered it a channel teachers could use to communicate their understanding of the course information to their trainers or supervisors, provide feedback and evaluate the application of their classroom practice. For example, student-teachers indicated (reflections):

Reflective writing is a useful way of professional development. Some teachers might try out new techniques and methods but never reflect upon the processes involved in the implementation or the consequences of applying these techniques. Teachers need to be trained as to how to improve their analytical and critical skills so that they can transfer this knowledge to their students.

Reflection is an important activity that should be in every single professional development program. Reflection could be as short as answering the questions or it could take a formal and structured form.

## Discussion

### Reflective practitioner designers of CPD courses

This study was conducted to explore the capacity of a research enquiry form developed by the author, called reflective practitioner designers of CPD courses, to support student-teachers in interpreting CPD theory and practice and developing an activist teacher professional identity. This investigation was achieved by analysing how student-teachers conceptualize definitions and factors constituting an effective CPD after completing reflective practitioner designers of CPD courses. In the discussion below, I discuss the findings in relation to the literature used in this study.

Regarding the research question, student-teachers used practitioner enquiry principles such as data collection, data reflection, framing intentions, and outcomes, identifying practical advantages for professionals or originators, administering enquiry, and concentrating the research on individual and group practices (Lunt & Shaw, 2015) to develop their CPD courses. They critically examined and reflected on their daily working conditions and routines (Murray, 1992) to develop teaching practices and promote an appealing school environment that encourages student learning (Stevens et al., 2016). Consequently, they identified the following issues to resolve: teaching English in large classes, EFL students’ speaking anxiety, improving students’ reading skills, lack of technological integration, providing feedback to students during classes, enhancing students’ thinking skills, integrating technology in providing feedback to students, assessing writing assignments for a classroom with a large number of students, the use of multiple mice presentation in EFL collaborative learning classrooms, and the gap between theory and practice in teaching grammar.

They then started designing their CPD course and were engaged in drafting their versions of CPD courses that would address their unresolved issues. This engagement made the experience vital and innovative (Hulse & Hulme, 2012); vital in the sense that they felt it is essential to find a solution to EFL teaching problems, innovative in the sense that their solutions should be creative but framed within the sociocultural factors found in teachers’ context, and most importantly applicable. In their reflections, they emphasized that CPD courses should meet EFL teachers’ needs and relate to what is occurring in real classrooms. This conclusion was reached after they actively engaged in CPD design and evaluation. Thus, their capacities to judge CPD courses were enhanced. This capacity was in line with Furlong (2011) and Cochran-Smitha & Lytleb (2004), who argued that teachers can enhance their practice by promoting specialist knowledge about their teaching and reinforcing knowledge in enquiry research focusing on practitioners’ knowledge gains. They shaped their knowledge of CPD and how these professional issues should be solved using different approaches to CPD. These approaches included aspects of professional learning (Bell & Gilbert, 1996), the capacity for professional autonomy and transformative practice supported by professional learning (Fraser et al., 2007), procedural knowledge acquisition and structured learning pedagogy (Kennedy, 2014), and the sphere of action in which professional learning takes place (Fraser et al., 2007).

In planning, most student-teachers preferred the transformative approach to CPD based on conceptual knowledge acquisition and transformative learning pedagogy. Their choice could be explained by their preference for knowledge construction methods in CPD courses. This preference is in line with Afshar & Doosti (2022); Bayram & Bıkmaz (2021); Uştuk & De Costa (2021), and Yalcin Arslan (2019), who claimed that professional development courses that were based on teachers’ active engagement were preferred by teachers and maximized their proficiency gains. Four student-teachers chose a transmission approach based on an administrated engagement of the trainee, such as workshops and coaching (see Table 1).

After completion, student-teachers reflected on the process of reflective practitioner designers of CPD courses. Their reflections involved the student-teachers’ reflection on their direct involvement in CPD course design and evaluation. Therefore, they defined CPD as a process of teaching an individual or a group of people to be successful and competent in their careers by improving their knowledge, skills, and their attitudes. Its goal is to increase students’ achievement by improving teachers’ or trainees’ pedagogical and content knowledge. It takes the shape of training workshops, communities of practices, monitoring, reflection, or action research derived from the teachers’ context.

In this study, student-teachers articulated the factors that constitute an effective CPD course. Regarding CPD planning, they revealed that in planning a CPD course, the following aspects should be focused on: teachers’ values, identities, and sociocultural factors found in their contexts. The knowledge presented should also be relevant and applicable in their classrooms. Furthermore, attending CPD courses should be optional. They believed that teachers should contribute to the planning of the CPD with their supervisors and head units of CPD. Finally, the content of the CPD courses should reflect the title of the course.

Regarding CPD pedagogy, through enquiry research, student-teachers drew attention to a new perspective of CPD, i.e., the trainer’s pedagogical practice. The trainer’s pedagogical practice and knowledge could be added to Bell’s and Gilbert’s (1996) professional learning aspects, including personal, social, and occupational. These factors may affect the success of professional development courses, and the implementation of its outputs (Fraser et al., 2007) and the trainer’s pedagogical practice used in CPD courses could be a fourth factor that may contribute to CPD courses’ success implementation of its outputs. Participants identified two main areas of pedagogical knowledge the trainer should have: knowledge of adult learning theories and teacher-driven approaches. In addition, they mentioned that presenters should have deep knowledge in the content area taught, have passion and empathy, be mindful, have substantive plans, practical presentation skills, and extensive knowledge on how to teach, design, and present workshops and be able to engage trainees actively. The trainers should also consider the teaching values and deeply understand the teachers’ context and needs. They emphasized that achieving high levels of perfection and confidence in the CPD classes’ content is impossible without a skilled and experienced trainer who models putting theory into practice. This knowledge generation, the trainer’s pedagogical practice, resonates with Torrance & Forde (2017), who claimed that practitioner enquiry could empower teachers to create a more extensive body of knowledge by generating context-specific understanding.

Finally, concerning CPD model and practice, participants argued that CPD courses based on reflection writing guided by transformative pedagogy are effective methods in teachers learning. They also argued for teachers to be involved in planning and setting the goals for their CPD courses. This argument is consistent with Afshar & Doosti (2022) who mentioned that to plan an effective CPD course; teachers should have the opportunity to select the course content and use interactive methods in content delivery, such as collaboration and collegiality among teachers.

### An activist teacher professional identity

Active practitioner designers of CPD courses contributed to the construction of an activist teacher professional identity developed by Sachs (2001). This contribution was achieved by adopting research-based enquiry that, according to Dickson (2011), facilitated the development of teachers’ understanding of the highly controversial relationship between practice, theory, and policy in teaching. This knowledge and understanding were evident in the capacity student-teachers demonstrated in planning and mapping CPD courses to meet their needs (see Table 1), their reflections on the definition of CPD courses, and the factors constituting effective CPD courses. They highlighted the main CPD principles and features for an effective CPD course. These principles reflected “equity and social justice” (Sachs, 2001, p.157), flexibility, and innovation in CPD courses design and delivery. They mentioned that CPD should consider EFL teachers’ needs, the institution’s needs, and the Saudi context and social norms. They considered teachers’ needs and paid attention to the organization’s needs and the Saudi context teaching norms. These principles indicated their sense of justice and equity for the rights of organizations, individuals, and the Saudi teaching context.

The participants’ flexibility and innovation over CPD practice were evident in their abilities to find agency and practice productive autonomy in educational contexts (Kennedy, 2014). Productive autonomy was practiced by extending the role of student-teachers beyond the traditional role: teachers merely receive knowledge in a top-down structure or via imposed CPD policies. Instead, they were active professional practitioners who constructed their versions of CPD courses. Their versions of CPD courses exemplified their flexibility and innovation in CPD theory and practice (see Table 1). They designed CPD courses that reflected their preferred CPD model and flexibility in planning CPD courses to meet their needs and address their teaching dilemmas. For example, in Table 1, one participant selected workshops and reflective writing to train teachers on how to assess writing assignments for a classroom with many students. Her CPD model used two pedagogical approaches to CPD courses: transmission and transformative. This CPD course planning indicated high levels of flexibility in delivering the CPD content to the trainees. The participant demonstrated a significant consideration of trainees’ learning preferences by using two approaches to deliver a CPD course.

In addition, participants outlined the essential pedagogical knowledge a CPD trainer should adopt. The trainer’s pedagogical knowledge and practice is an area of knowledge rarely discussed in CPD literature or considered in practice. However, they discussed the trainer’s knowledge in their reflections. Such innovation in CPD theory and practice is what student-teachers need to acquire an activist teacher professional identity. Consequently, after graduation, they can create their CPD communities based on their values and beliefs of what a CPD course should be, liberating them from the control of organizations over CPD courses. Therefore, they may resist neoliberal and managerial practices that curtail teacher autonomy (Kennedy, 2014). Ultimately, autonomous, active teachers will have more confidence, improve teaching discipline, and increase student achievement.

## Conclusion

The author and student-teachers dealt with CPD as a science to explore and understand. This research paper contributes to the body of literature in light of two main areas of knowledge. First, teaching and training teachers in CPD principles and theories, the objective is to liberate them from the strains of the new bureaucratic, managerial movement in education that controls what teachers learn, thereby creating agents for change among teachers responsible for their CPD journeys. Second, MA teaching courses could develop new venues and trends for enquiry practitioner research to examine and investigate the needs of their students for more targeted and effective teaching skills.

This study has a few limitations. For instance, the population sample only comprises ten female student-teachers because only eleven student-teachers enrolled in this course. Future studies could apply this intervention to many students and teachers of both genders.

Student-teachers in this study provoked pedagogical knowledge of CPD, and future research studies could address this area of research. They could discuss how trainers in charge of CPD units in institutions, schools, and universities plan and present an effective CPD course. This investigation could be achieved by expanding on their educational orientations towards CPD, the teaching methods used in their CPD programs, and the reasons behind these choices and preferences.

## Data Availability

The data that support the findings of this study are not openly available due to the agreement stated in the consent form and signed by the participants that their identity will remain anonymous.

## List of abbreviations

TESOL: Teaching English to speakers of other languages.
EFL: English-as-a-Foreign-Language.
CPD: Continuous professional development.
PBE: Practitioner Based Enquiry.
